# Educational achievement among children with a disability: do parental resources compensate for disadvantage?

**DOI:** 10.1016/j.ssmph.2023.101465

**Published:** 2023-07-17

**Authors:** Idunn Brekke, Andreea Alecu, Elisabeth Ugreninov, Pål Surén, Miriam Evensen

**Affiliations:** a Department of Childhood and Families, Division of Mental and Physical Health, Norwegian Institute of Public Health, Norway; b Consumption Research Norway, Oslo Metropolitan University, Oslo, Norway; c Norwegian Social Research, Oslo Metropolitan University, Oslo, Norway; d Department of Child Health and Development, Division of Mental and Physical Health, Norwegian Institute of Public Health, Norway; e Institute for Social Research, Norway

**Keywords:** Child disability, Diagnosis, Socioeconomic status, Educational performance, GPA scores

## Abstract

We examined the impact of child disability on Grade Points Average (GPA) using all children aged 15–16 years who completed their lower secondary education and registered with a GPA score in the period from 2016 to 2020 in Norway (n = 247 120). We use registry data that contain information on the child's main diagnosis, such as physical-, neurological- and neurodevelopmental conditions, and the severity of the condition, additional to the child's family characteristics. First, we examined whether the impact of the child's disability on the GPA scores varied by diagnosis and the severity of the child's condition. Second, we examined whether higher parental socioeconomic status (SES) buffers against the negative impact of child disability on GPA scores. Using longitudinal register data with the school fixed-effect model, the results showed that children with neurological and neurodevelopmental disabilities obtained lower GPA scores than their typically developing peers without chronic conditions, however children with asthma and diabetes had comparable GPA scores. These associations were most evident for neurodevelopmental conditions, such as ADHD and autism but also notable for neurological conditions such as epilepsy. In general, a severe condition impacts GPA scores more negatively than a less severe condition. Moreover, our analysis revealed that children of highly educated parents obtained higher GPA scores than children who had parents with short education. This applied to both disabled and typically developing peers, except children with autism and epilepsy, among whom buffering due to the parent's education did not seem to apply.

## Highlights

• Children with a disability obtain lower GPA scores than their typically developing peers without chronic conditions.
• These associations are most evident for neurodevelopmental conditions.
• A severe condition impacts GPA scores more negatively than a less severe condition.
• Children of highly educated parents obtain higher GPA scores than those of lower educated parents.

## 1 Introduction

Education for all has long been a basic principle, and it is a stated policy goal that children should have equal rights to education, regardless of gender, social and cultural background and any special needs (Universal Declaration of Human Rights). Still, there are large inequalities in educational achievement and attainment due to socioeconomic factors (Breen & Jonsson, 2005; Broer et al., 2019). Moreover, children with a disability continue to face challenges in school and they (Rivera and Tilcsik, 1224), experience worse educational outcomes than their typically developing peers (Eide et al., 2010; Esch et al., 2014; McKinley Yoder & Cantrell, 2019). The evidence further suggests that the association between child disability and educational outcomes is generally strongest for mental disabilities (Mikkonen et al., 2020; Nordmo et al., 2022).

There are strong socioeconomic inequalities in the prevalence of child disabilities (Blackburn et al., 2010; Carrilero et al., 2021; Reiss, 2013). However, there is little research on how the impact of child disability on educational outcomes varies according to socioeconomic status (SES) (Chatzitheochari & Platt, 2019). The family into which a child is born is of great importance since that determines the resources available to the child. Unequal access to family resources (i.e. social, cultural and economic capital) in childhood may create further differences in educational disparities between disabled and typically developing children. Moreover, social and physical barriers at school may also impair disabled children's educational attainment and therefore important to take into account (Shandra & Hogan, 2009). The relatively few studies that have examined the extent to which family SES moderates the relationship between child disability and educational outcomes (i.e educational achievement and attainment) have shown mixed findings. Some studies show that the negative relationship between child disability and educational outcomes varies by parental SES (Jensen et al., 2021; Mikkonen et al., 2020), while other studies conclude that the negative relationship between child disability and educational outcomes does not vary by parental SES (Mikkonen et al., 2020; Evensen et al., 2016; Currie & Stabile, 2007; von Simson et al., 2020).

This study contributes to the literature on inequalities in educational attainment among children with disabilities by exploring how type of disability and parental SES jointly affect children's Grade Points Average (GPA) scores. To address this issue, we use register data including five full cohorts (2000–2004) of children in Norway (n = 247 120), with information on the child's main diagnosis, the severity of the condition, additional to GPA scores and the child's family characteristics. To examine whether parental SES affect the GPA scores among disabled children, we restrict our sample to children attending ordinary schools and compare different groups of children by using diagnosis and severity of the child's condition combined with high or low parental SES. To identify how parental SES and disability are related to GPA scores, we compare with the GPA score to children from high and low parental SES without disabilities. We employ an analogue comparison of children with and without disabilities for each of the groups included.

## 2 Theoretical background, hypothesis and previous research

Several studies have examined the impact of child disability on educational outcomes. Generally, the results show that children with a disability have worse educational outcomes than typically developing children. This is found in the United States (Breslau et al., 2008; Champaloux & Young, 2015), Canada (Duncan et al., 2021), United Kingdom (Lereya et al., 2019) and elsewhere in Europe (Agnafors et al., 2021; Evensen et al., 2016; Mikkonen et al., 2020; Rasalingam et al., 2021; Veldman et al., 2014). There are many reasons why children with a disability have poorer educational outcomes than their typically developing peers. School absenteeism, reduced cognitive functioning, reduced aspirations and a lack of family resources are discussed in the literature (McKinley Yoder & Cantrell, 2019). Children with a disability may experience pain, fatigue and personal challenges that significantly impact their school life and lead to school absenteeism (Mizunoya et al., 2018). Some of these children also have conditions that require medical follow-ups and treatment that cause them to miss days at school. There is robust evidence that school absenteeism is detrimental to children's educational achievement (Klein et al., 2022), and school absenteeism is also known to be socioeconomically stratified (Sosu et al., 2021). Structural barriers in different ways, depending on the child's condition are also found to contribute to educational inequalities between disabled and typically developing children (Chatzitheochari & Platt, 2019; Shandra & Hogan, 2009). A recent review highlight that there is a lack of support (e.g. material, technical, and training) for the pedagogical staff, and that the teachers need more preparation to work in the inclusive classroom (Kurowski et al., 2022). Social and physical barriers exists for individuals with disabilities, particularly those with severe disabilities, and it is documented that the severity of a child's condition is important for educational outcomes, where those with the most severe condition are found to be the most impaired (McKinley Yoder & Cantrell, 2019). Therefore, we hypothesize that children with a disability have poorer GPA scores than typically developing children without chronic conditions and that children with a severe condition are particularly at risk of poor GPA scores.

Generally, there is evidence that both physical disability (McKinley Yoder & Cantrell, 2019) and mental disability (Melkevik et al., 2016) have negative impacts on educational outcomes. In particular, children with externalizing problems seem to have a higher risk of poor educational outcomes (Esch et al., 2014; Evensen et al., 2016). Moreover, the link between child disability and educational outcomes seems to be stronger for mental health conditions than for physical conditions and applies only to some types of physical disabilities (Currie & Stabile, 2006; Mikkonen et al., 2020; Nordmo et al., 2022). Sometimes, a child's condition will only affect the child's physical functioning, and some of these conditions may be treated with medication that works well and may therefore not impact school performance. Some diagnoses influence the child's cognitive and social function and the ability to concentrate, which makes it difficult for the children to socialize and learn in school. Therefore, the link between child disability and educational achievement will most likely vary by type of diagnosis. In the present study, we examined children with physical conditions (i.e. asthma and diabetes), neurological conditions (i.e. CP and epilepsy) and neurodevelopmental conditions (i.e. ADHD, autism and Asperger's). These diagnoses impact children in different ways, which in turn will affect their educational achievement differently.

Previous research shows that asthma commonly results in school absenteeism (Fleming et al., 2019a) due to attacks or difficulty with breathing, night cough and bronchitis. However, consensus is lacking on whether this translates into impaired educational outcomes (Schneider, 2020). Some studies found that children with asthma performed worse in school-based assessments (Fleming et al., 2019a; Mitchell et al., 2022; Nilsson et al., 2018; Senter et al., 2021), while other studies found little to no difference in their school performance compared to peers without asthma (Crump et al., 2013; Lundholm et al., 2020). A recent study that examined a variety of different childhood diseases shows that there was no educational burden related to child asthma (Nordmo et al., 2022). The evidence regarding the impact of type 1 diabetes on educational outcomes is also mixed. Some studies have found that children with type 1 diabetes have poorer educational outcomes compared with their healthy peers (Fleming et al., 2019b; Persson et al., 2019), while other studies show negligible differences in the educational outcomes of children with and without type 1 diabetes (Begum et al., 2020; Cooper et al., 2016). Several studies document that children with epilepsy have higher school absenteeism, are more likely to have special educational needs and have lower educational attainment than their typically developing peers (Champaloux & Young, 2015; Fleming et al., 2019c). Comparable results are found for children with CP (Gillies et al., 2018); however, much of the educational impairment among children with CP can be explained by intellectual disability (Jarl & Alriksson-Schmidt, 2021).

Attention-Deficit/Hyperactivity Disorder (ADHD) is one of the most common neurodevelopmental disorders among children. Symptoms of ADHD include inattention, hyperactivity, and impulsivity, which can lead to problems at school. Previous research has documented that ADHD is associated with negative school achievement and attainment (Arnold et al., 2015; Currie & Stabile, 2006; Nordmo et al., 2022; Sunde et al., 2022). Sunde et al. (Sunde et al., 2022) document that children with ADHD have significantly lower GPA scores compared with those without ADHD. This also applies when comparing siblings. Moreover, treatment with ADHD medication seems to have a positive correlation with educational achievement (Jangmo et al., 2019; Keilow et al., 2018).

Children with autism spectrum disorder (ASD) often have problems with social interaction, empathy, communication, and flexible behaviour. The severity of the disability and the combination of symptoms vary greatly from child to child. Problems in executive functioning among youth with ASD play an important role in academic progress (Dijkhuis et al., 2020), and under-achievement among students with ASD has been reported compared with typically developing peers in several studies (Ashburner et al., 2010; Keen et al., 2015; Kim et al., 2018).

In summary, there is some indication that mental disabilities are more detrimental to educational outcomes than physical disabilities. Thus, we hypothesize that neurodevelopmental disabilities in childhood impact GPA scores more negatively than neurological and physical disabilities.

While disability may affect children's physical and mental capacities for learning in and of itself, family resources may also play a role. Family resources refers to a range of different resources such as economic, social, cultural, cognitive, physical, and community resources. Family SES is a measure of children's’ access to these family resources and thus reflect the economic, social and human-capital resources available to the children (Improving the measurement of socioeconomic). For example, there are reasons to expect that parental response and involvement in a child's disability might depend on the socioeconomic circumstances of families. High-SES parents have more social, economic and educational resources to provide the type of schooling that is beneficial to their children (Lareau, 1989). Resources are differently distributed in families, and these differences have an impact on how and in which ways parents invest in their children (Parcel et al., 2010). Parents invest in their children with both time and money. Parental education is an important predictor of children's cognitive skills development (Biedinger, 2011; Noble et al., 2015). There is a large body of literature examining socioeconomic differences in parent–child interactions. Studies that examine parental time use show that highly educated parents spend more time with their children, and they use more time on developmental activities (i.e. reading, playing, talking to and teaching) than do less educated parents (Guryan et al., 2008). Highly educated parents are more likely than their less educated counterparts to support and encourage child exploration, and they are in general more involved in their children's education (Cheadle & Amato, 2010; Lareau, 2011).

Family SES may also determine the types of investment strategies parents can afford (Conley & Lareau, 2008). A family's economic resources are found to be important for a range of outcomes in children, including cognitive and social–behavioural development and health (Cooper & Stewart, 2021). Economic resources may be critical for the possibility of assisting a child with a disability to attain an education. Low-income families may struggle to pay for facilities and assistance that make everyday life easier for children with disability (van der Mark et al., 2017) so that they are more able to focus on their schoolwork. Moreover, parents with better socioeconomic resources in terms of income and education are in a better position to negotiate with public services in their children's interest (Cooper & Stewart, 2021).

Compensatory advantage theory emphasize that social inequalities can be explained by the interaction of individual characteristics, such as disabilities and social structures (Bernardi, 2012, 2014; Bernardi & Grätz, 2015; Huang, 2020). Compensatory advantages arise when individuals can leverage their advantageous characteristics to overcome disadvantages despite the barriers they face. For example, highly educated parents may be able to use their education and skills to secure that their disabled children get the resources they need in school and the larger society alike. Moreover, parents with high income may also be able to live in neighbourhoods with good-quality schools that attempt to give all children opportunities for a good quality education. Several empirical studies support the compensatory advantage theory [e.g. (Conley & Lareau, 2008), (Bernardi, 2014), (Hsin, 2012), (Almond & Mazumder, 2013), (Bernardi & Triventi, 2018)]. For example, Conley and Lareau (Conley & Lareau, 2008) report that highly educated mothers tend to compensate for endowment differences among their children by spending more time with the child who has the worst health (measured as low birth weight). Opposite results were uncovered for lower educated mothers.

According to compensatory advantage theory, the adverse effect of child disability on educational outcomes may be reduced in high-SES families and worsened in low-SES families. With our compensatory advantage hypothesis, we hypothesize that high-SES families compensate for children's disabilities in GPA scores. If disabled children from high-SES households have GPA scores comparable to those of their typically developing peers without chronic conditions from high-SES households, we defined this as full compensation. Moreover, if disabled children with high parental SES have GPA scores comparable to those of their typically developing peers without chronic conditions from low-SES households, we defined this as part compensation.

### 2.1 The school system in Norway

At the beginning of the 1960s, the nine-year primary school was introduced in Norway, and the process of enrolling disabled children from special schools or special classes into public regular schools started. The educational system in Norway consists of primary school, lower secondary school and upper secondary school. Elementary and lower secondary schools are mandatory for all children aged 6–16, and public education in Norway is free of charge. All children between the ages of 16 and 19 have a statutory right to three years of upper secondary education. Upper secondary school is not mandatory; however, 98% of youths enrol in upper secondary education immediately after lower secondary school (The Norwegian Directorate for Education and Training, 2019). The school system in Norway is characterized by late tracking – the students' progress through the same system until the age of 16, after which they must choose between various academic or vocational programmes. Hence, the lower secondary GPA is the first grade that may impact students’ future academic endeavours.

Inclusive education is a fundamental principle in Norwegian primary and secondary education. This implies that all children learn together in mainstream schools regardless of whether they have a disability or special educational needs. Children who do not get a satisfactory result from the ordinary education have rights to get adapted teaching at their school received in the corresponding class, in groups or alone with an assistant or teacher. The right to special education or adapted teaching, is not linked to a diagnosis or difficulty, but to the pupil's lack of benefit from the ordinary education (Mathiesen & Vedøy, 2012).

For a school to be inclusive, it must organize and adapt learning for all students. All students should have the opportunity to learn in a way that is adapted to their talents and abilities (Kurowski et al., 2022). The idea of inclusive education is based on the social model of disability. This is a way of understanding that people are disabled by barriers in society rather than being different or having impairments. The medical model on the other hand assumes that the difficulties faced by the disabled child are a direct result of their individual disability. Thus, the way a teacher with a medical model orientation versus a teacher with a social model orientation influences how the they interact and work with a child with a disability in school (Haegele & Hodge, 2016), which in turn may have consequences for the child's educational outcomes.

## 3 Data and methods

### 3.1 Study setting and sample

Our sample consists of all children aged 15–16 years who completed their lower secondary education and registered with a GPA score in the period from 2016 to 2020 in Norway (n = 247 120). In this study, we use information from several linked administrative register data sources: the National Educational Database (NUBD) and the Historical Event Database (FD-trygd) administered by Statistics Norway, which includes rich longitudinal population data about income and wealth, welfare benefits, employment, education and demographic information linking parents and children. Children with a disability are identified using information on attendance benefits derived from FD-trygd. Children who need long-term private care and supervision because of a medical condition are entitled to attendance benefits from the Norwegian Labour and Welfare Administration (NAV). Attendance benefit is a non-means-tested cash benefit adjusted to the severity of increased care needs for which parents need to file an application. The care needs must last for two to three years or more. The benefit is paid at four different rates, reflecting mild to severe care needs. The overall workload of the person providing the care/supervision is the determining factor^1^. We also use information on the main diagnosis linked to attendance benefits obtained from the NAV register. Diagnostic codes are recorded in the NAV register, according to the World Health Organization's International Classiﬁcation of Diseases, version 10 (ICD-10). All data sources utilized in this study are administered and merged by Statistics Norway. Data linkage is facilitated by the unique ID number assigned to all residents of Norway. The study was approved by the Regional Committee for Medical Research Ethics in South-East Norway. The Norwegian Data Protection Authority granted permission to access all the databases and records without written consent from the study participants.

### 3.2 Measures

The dependent variable in this study is *GPA scores*, which are measured in the 10th grade as an average of credits/marks reflecting performance in class, tests and national exams in all 11 main school subjects undertaken in primary school. Grades vary between 1 (low) and 6 (highest). The GPA is calculated by adding together all the final grade averages and exam and dividing by the number of grades. Then the average is multiplied by 10; hence, the GPA varies between 10 and 60. Exemptions from GPA scores may be approved for students with disabilities. Therefore, it may be that children with the most severe conditions do not have GPA scores (see Supplementary Appendix-Table S1). The main independent variable (exposure variable) is *child disability*. Children with a disability are identified using information on attendance benefits and associated diagnoses. Children who receive attendance benefits before receiving their GPA (which is at 16 years of age) are classified as children with a disability or impairment. The attendance benefit is paid at four different rates, reflecting mild to severe care needs. We use paygrade as a proxy for the *severity of the child's disability*, ranging from grade 1 to grade 4. We differentiate between paygrade 1 (1 = low attendance benefit) and paygrades 2 to 4 (2 = high attendance benefit), and the reference group is typically developing children without chronic conditions = 0 (i.e., children not registered with attendance benefits or basic benefits in FD-trygd). For children receiving attendance benefits, we have information on their main diagnoses. This also implies that we do not have data on comorbidities or additional diagnoses. We distinguish between *neurodevelopmental, neurological and physical conditions*. Neurodevelopmental conditions include attention-deficit hyperactivity disorder (ADHD) (ICD-10: F90), autism spectrum disorder (ASD) and Asperger's syndrome (ICD-10: F84.0, F84.1 and F84.5). Physical conditions include type 1 diabetes (ICD-10: E10) and asthma (ICD-10: J45 and J46). Neurological conditions include epilepsy (ICD-10: G40), cerebral palsy and other paralysis syndromes (ICD-10: G80-G83). We classified the remaining diagnoses as “Other diagnoses”, which include a range of different disorders, as well as children who receive attendance benefit but have no registered diagnosis. Independent variables used in the model were selected based on findings in the literature (Mikkonen et al., 2020) and data available in the register data.

Socioeconomic status (SES) is measured by parental education and parental income. We measure the highest parental education among biological parents (i.e., either mother's or father's education) at age 10*. Parental education* is divided into two levels: 1 = bachelor's degree level and above, 0 = upper secondary school and lower. *Parental income* is measured as the total of both parents' combined incomes, measured at age 10. This includes salary and income from self-employment. Parents' income is measured in Norwegian kroner, and we use the yearly median income as the cut-off (1 = <median income, 0 = ≥ median income). The age of the mother at birth is measured in years, but to allow for non-linearity, we included age-squared in the model. Immigrant background is measured as follows: 1 = two parents born outside Norway, 0 = at least one parents born in Norway. Marital status was coded as follows: 0 = unmarried, 1 = married or living with a partner, 2 = divorced or separated. Parental divorce was measured as parental divorce before the child turned 10 years old. The number of siblings was indicated by three dummy variables: 1) single child, 2) two children and 3) three or more children. The gender of the child is coded as follows: boys = 0 and girls = 1. We also control for *birth cohort*. In the register data, we can identify which schools the children attend, and thus, we can control for the characteristics of children's schools (and neighbourhoods) using school fixed effects.

### 3.3 Statistical methods

**Fig. 1:**
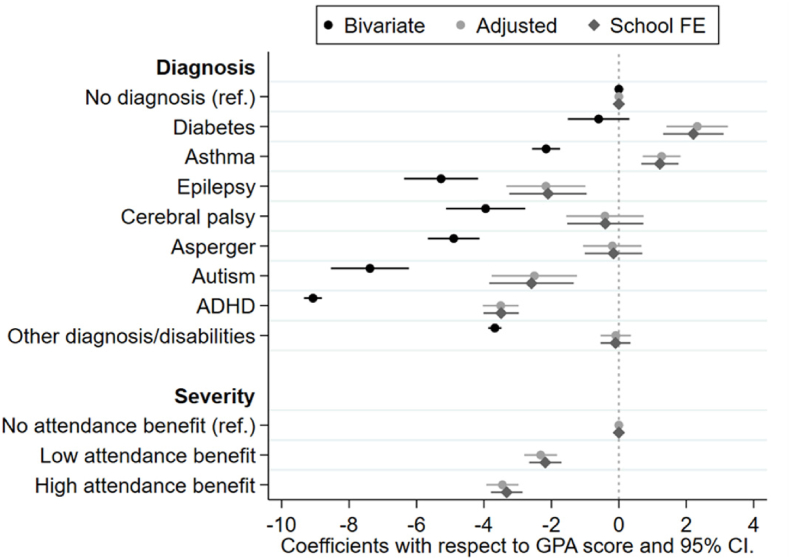
Linear prediction of GPA scores dependent on diagnosis, severity and parental SES, (OLS regression). Note: Model 1 (bivariate) shows the bivariate correlation between child diagnosis and GPA scores, Model 2 (adjusted) adjust for severity of the child's diagnosis, child gender, immigrant background as well as parental SES, mother's age at birth, marital status and number of siblings. In Model 3 (School FE) we add school fixed effects. All models control for birth cohort.

**Fig. 2:**
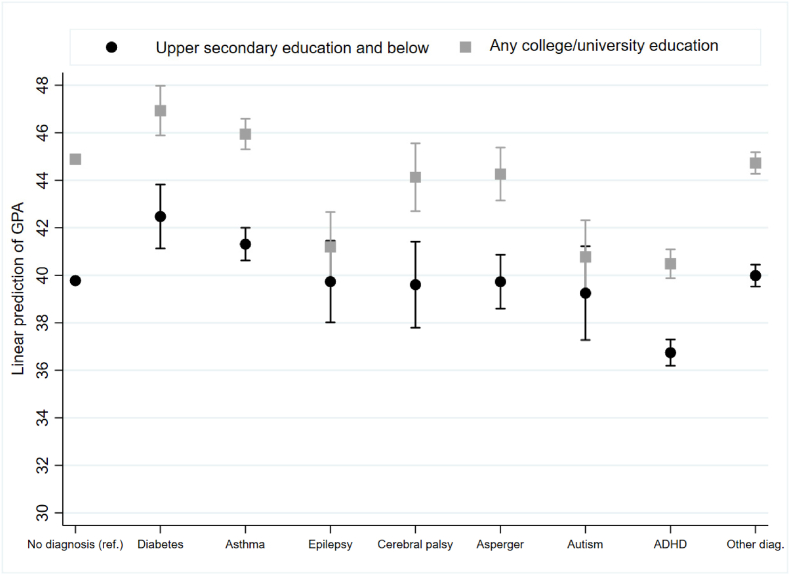
Linear prediction of GPA scores based on the interaction between parents's educational level and children's diagnosis, (OLS regression), based on model with full controls and school FE

**Fig. 3:**
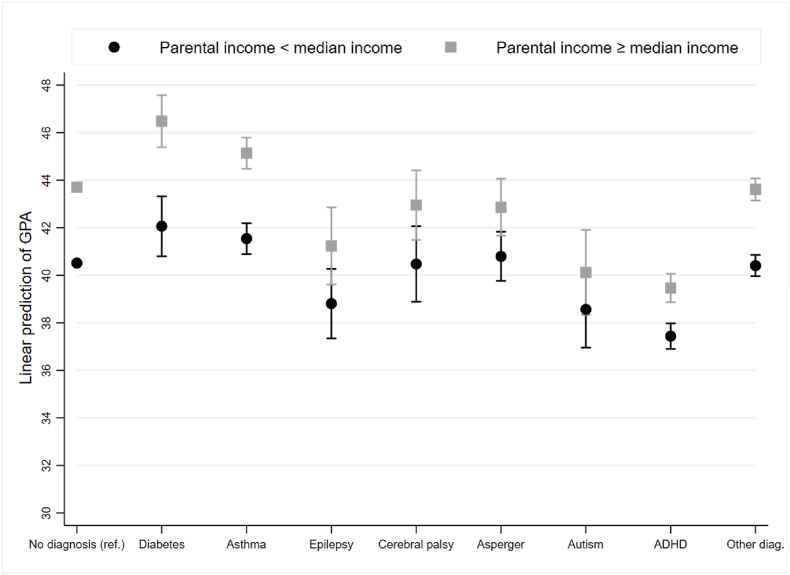
Linear prediction of GPA scores based on the interaction between parental income and children's diagnosis, (OLS regression), based on model with full controls and school FE

Descriptive analyses were presented with means (SD) and proportions (%). For the analysis of GPA scores, we used ordinary linear regression analyses (OLS) with robust standard errors. First, we estimated the linear prediction of GPA scores for children diagnosed with neurodevelopmental, neurological and physical disabilities compared with typically developing peers without chronic conditions in three different models (Fig. 1). Model 1 shows the bivariate correlation between child diagnosis and GPA scores. Model 2 adjusts for severity of the child's diagnosis, child gender, and immigrant background as well as parental education, parental income, mothers age at birth, parental divorce, and number of siblings. In Model 3, we added school fixed effects. Moreover, to investigate whether parental SES modifies the relationship between child disability and GPA scores, we introduced the interaction term between parental education and child diagnosis (Fig. 2). Finally, we introduced the interaction term between parental income and child diagnosis (Fig. 3). To take confounding factors at the school level into account, we included school fixed effects in both Fig. 2, Fig. 3. Statistical analysis was performed using STATA® 17, and the statistical significance level was set to p < 0.05.

## 4 Results

### 4.1 Descriptive analyses

Our descriptive analysis showed variation in GPA scores by type of diagnosis, where the lowest GPA scores were found among children with ADHD and autism (see Supplementary Appendix-Table S2). Children with diabetes and asthma had, on average, GPA scores comparable to those of typically developing peers without chronic conditions, while children with epilepsy, CP, Asperger's, autism and ADHD had lower GPA scores than their typically developing peers without chronic conditions, particularly children with ADHD and autism. Parental education and parental income were lowest among children diagnosed with ADHD and highest among typically developing peers without chronic conditions. The distribution of the GPA scores (density plot) by diagnosis is shown in Supplementary Appendix-Fig. S1).

### 4.2 Child disability and GPA scores

Fig. 1 presents the linear prediction of GPA scores (with 95% Cls) for different child disabilities on GPA scores (the figure is based on Supplementary Appendix-Table S3). Model 1 only includes child diagnosis and thus the coefficients show the bivariate associations between child diagnosis and GPA scores. In the bivariate model, children with diabetes had GPA scores comparable to those of typically developing peers without chronic conditions. Children diagnosed with asthma had, on average, 2.0-point lower GPA scores than typically developing peers without chronic conditions. Children with epilepsy and CP had, on average, 4.9- and 3.7-point lower GPA scores than their typically developing peers without chronic conditions. The comparable numbers for children with Asperger's syndrome, ADHD and autism were 4.6, 8.7 and 7.1 points, respectively. These initial results support the claim that child disability is an important predictor of educational performance. Adjusting for the severity of the child's diagnosis, gender and immigrant background, family SES and other characteristics of the mother (i.e., age at birth, parental divorce, number of children) reduced the associations substantially, but significant associations remained for epilepsy, autism and ADHD. Children with epilepsy, autism and ADHD had, on average, 2.1-, 2.5- and 3.5-point lower GPA scores than their typically developing peers without chronic conditions in the adjusted model. Children diagnosed with asthma and diabetes had somewhat higher GPA scores compared with typically developing peers without chronic conditions in the adjusted model. For children with CP, Asperger's and “other diagnosis”, we did not find any significant differences in GPA scores compared with typically developing peers without chronic condition. Moreover, children who receive low attendance benefits had, on average, 2.3-point lower GPA scores than typically developing peers without chronic conditions who receive no attendance benefits, and the comparable number for children with high attendance benefits were 3. These results supported the claim that the severity of a child's condition is a significant predictor of educational performance. Further, the inclusion of school fixed effects (controlling for all stable characteristics of the children's schools) did not change the observed association. The results revealed that neurodevelopmental disabilities, such as autism and ADHD, as well as epilepsy, which is a neurological disorder, seem to be more detrimental to GPA scores than physical disabilities and that the severity of the child's condition seems to be an important determinant of GPA scores.

### 4.3 Child disability, parental SES and GPA scores

Fig. 2 presents the linear prediction of GPA scores based on the interaction term between parent's educational level and children's diagnosis (the figure is based on Supplementary Appendix-Table S3, model 4). In predicting GPA scores, we examined the extent to which parental education compensates for the child's disability. Children of highly educated parents obtained higher GPA scores than children of who had parents with short education. This applied to both disabled and typically developing peers without chronic conditions, except for children with autism and epilepsy, who had similar GPA scores regardless of the parent's educational level.

Comparing children of parents with any college/university education, children with CP and Asperger's obtained comparable GPA scores compared to typically developing peers without chronic conditions. This result demonstrated that with all other variables held constant, children diagnosed with CP and Asperger's fully compensated for their disability if they had highly educated parents. Moreover, Fig. 2 showed that children with type 1 diabetes, asthma and “other diagnosis” had GPA scores that are higher than or comparable to those of typically developing peers without chronic conditions.

Children with ADHD born to parents with a long education obtained GPA scores comparable to those of their typically developing peers without chronic conditions of parents with a short education. This result revealed that children diagnosed with ADHD partly compensated for their disability if they had highly educated parents since they achieved comparable GPA scores as their typically developing peers without chronic conditions of parents with short education. For children with epilepsy and autism, long parental education does not seem to impact GPA scores. However, children with epilepsy and autism, regardless of their parent's educational level, obtained GPA scores comparable to those of typically developing peers without chronic conditions with short education.

Fig. 3 presents the linear prediction of GPA scores based on the interaction between parental income and children's diagnosis (the figure is based on Supplementary Appendix-Table S4). We used median income as a cut-off. In additional analyses, we used three, four- and five-income groups. These analyses showed comparable results, which strengthens the robustness of our results. Children from households of parents with above-median income had, on average, higher GPA scores than those from households of parents with below-median income. This applied to typically developing peers without chronic conditions and children with diabetes, asthma, ADHD and other diagnoses. However, we found no such differences among children diagnosed with epilepsy, CP, Asperger's, and autism. Moreover, Fig. 3 show that children with CP and Asperger's born to parents with income above the median had GPA scores comparable to those of typically developing peers without chronic conditions of parents with above the median income. This result demonstrated that with all other variables held constant, children diagnosed with CP and Asperger's fully compensated for their disability if they had parents with income above the median. Children diagnosed with ADHD born to parents with income above median obtained, on average, GPA scores lower than those of typically developing peers without chronic conditions of parents with income below median. Parental income does not seem to protect against ADHD deficits in the prediction of GPA scores.

We have also tested the interaction between child gender and diagnosis. The results showed that girls obtain higher GPA scores than boys, except for children with Asperger's and autism, where we did not find any significant gender differences.

## 5 Discussion

The objective of the current study was twofold. The first was to explore the relationship between a child's physical-, neurological- and neurodevelopmental conditions and its GPA scores. Secondly, to examine if higher parental SES works as a buffer against the potential negative impact on GPA scores that a child's disability may have. Studies in the public health field mainly focus on the impact of the child's medical condition per se and, to a lesser extent, examine the interplay between the child's medical condition and family resources. At the same time, studies in the field of sociology often lack information on the child's diagnosis and the severity of the child's medical condition. To bridge the gap between studies examining educational performance among children with a disability in sociology and public health, we draw on theoretical developments in the stratification literature and illustrate their application to the public health literature and vice versa. In the present study, we have used information on the child's diagnosis and severity, and in addition, we have examined the interplay between the child's medical condition and parental SES.

Using longitudinal register data with the school fixed-effect model, we find that children with a disability obtain lower GPA scores than their typically developing peers without chronic conditions, except for children with diabetes, who have comparable GPA scores. These associations are most evident for neurodevelopmental conditions but are also notable for neurological conditions. The results also indicate that a severe condition impacts GPA scores more negatively than a less severe one. Furthermore, from the starting point that children with a disability have substantially lower GPA scores than typically developing peers without chronic conditions, if we adjust for the severity of the child's diagnosis, it seems that family SES and other characteristics of the family substantially moderate these associations. No significant association was found between disability and GPA scores among children diagnosed with asthma, CP, and Asperger's. This indicates that differences in individual and family factors could explain the association uncovered for children with asthma, CP, and Asperger's in the unadjusted model. However, significant associations remain for children with epilepsy, autism, and ADHD, which implies that these conditions have a negative correlation with GPA scores regardless of the severity of the child's condition and family SES. The associations are robust when controlled for school-level factors, suggesting that the remaining association is not due to the sorting of children into different schools.

Our further analysis revealed that children of highly educated parents obtain higher GPA scores than children who had parents with a short education. This applies to both disabled and typically developing peers without chronic conditions, except for those with autism and epilepsy to whom a buffering due to the parent's education does not seem to apply. A potential explanation for this may be the fact that many children with epilepsy and autism have cognitive impairments and suffer from learning disabilities (Beghi et al., 2006; O'Brien & Pearson, 2004) in such a way that they need special education in school. Thus, in terms of school achievements, highly educated parents may also struggle to compensate for the child's impairment if the disability is combined with either autism or epilepsy, and if special educational support is required. In the same vein, previous research indicates that low-SES students are more likely to receive special education than high-SES students (Kvande et al., 2018). Special education is a school-based activity that helps reduce social inequality by providing appropriate learning opportunities for all children (Hibel et al., 2019). This could help explain why we do not find any differences in GPA scores between low- and high-SES students diagnosed with epilepsy and autism.

Whereas many children with autism have cognitive impairment, children with Asperger's syndrome do not suffer cognitive delay and will normally have cognitive functioning in line with other children (Frith, 2004). Still, children with Asperger's often struggle with peer relationships and socialization, which may affect educational performance, although previous research indicates that children with Asperger's can learn to socialize adequately (Koegel et al., 2012). According to Lareau (Lareau, 1989), there are reasons to believe that highly educated parents—to a greater extent than parents with short education—can use their social capital to promote their child's socialization and school functioning and thus compensate for its disability. In line with this, we found that for children with Asperger's and CP, high parental education seemed to fully compensate for the child's disability in predicting GPA scores.

Our results show that children diagnosed with ADHD born to parents with short education stand out with particularly low GPA scores. Children with ADHD born to parents with a long education obtain GPA scores comparable to those of their typically developing peers without chronic conditions born to parents with a short education. This result may reflect the fact that highly educated parents are able to compensate for some of the negative impacts of ADHD. High-SES parents may be better at manoeuvring schools and the health care system to make sure that their child gets appropriate treatment and support and avoids negative stigma (Blum, 2015; Lareau, 1989). Previous research has also found that children from families with fewer resources often experience more health problems and are less able to recover from health shock than those from high-SES families, exacerbating socioeconomic disparities in child disability (Currie & Stabile, 2003). In our study, comparable results are found for the analysis of parental income and GPA scores, but parental education has a somewhat stronger impact than parental income. Our results are in line with previous research showing a larger negative impact of neurodevelopmental conditions than physical conditions on educational performance (Currie & Stabile, 2006; Mikkonen et al., 2020; Nordmo et al., 2022). In line with these studies (Begum et al., 2020; Cooper et al., 2016; Nordmo et al., 2022), our study validates the conclusion that children with diabetes perform on the same level or even higher than typically developing peers without chronic conditions. Moreover, our results show that children with asthma have GPA scores comparable to those of typically developing peers without chronic conditions when comparing children with the same family SES. Similar findings were made by Crump et al. (Crump et al., 2013), Nordmo et al. (Nordmo et al., 2022) and Lundholm et al. (Lundholm et al., 2020). This result may reflect the fact that in Norway, children diagnosed with diabetes and asthma seem to receive adequate treatment so that the children's disease does not impact their school attendance and performance.

In line with numerous other studies, our study reveals that children diagnosed with ADHD, autism and epilepsy obtain lower GPA scores than typically developing children, even after adjusting for family SES. We found that children with ADHD had substantially lower GPA scores compared with typically developing peers without chronic conditions and stand out as the group with the lowest GPA scores. Our results align those of Jangmoe (Jangmo et al., 2019), Nordmo et al. (Nordmo et al., 2022) and Sunde (Sunde et al., 2022), who reported a large ADHD deficit in educational performance using register data from Sweden and Norway. We also found that children with autism and epilepsy had lower GPA scores than typically developing peers without chronic conditions, which is in line with previous research findings (Ashburner et al., 2010; Champaloux & Young, 2015; Fleming et al., 2019c; Keen et al., 2015; Kim et al., 2018; Nordmo et al., 2022).

The present study has several strengths, including a large sample size (n = 247 120), rich longitudinal register data, a wide range of sociodemographic information and an objective measure of child disability, ruling out any reporting bias. Another strength of this study is the inclusion of data on children with different types of neurodevelopmental, neurological and physical conditions, which made it possible to analyze variations in GPA scores between diagnosis groups. Our study also has some limitations. First, we identified children with a disability only if they were administratively recognised as such and received assistance allowance, which does not encompass all children with a disability and may underrepresent those with less severe conditions. For some diagnoses, the proportion that have exempt from GPA is high. This implies that we most likely have a sample with the most well-functioning children that have registered GPA scores. Second, the diagnoses recorded in the NAV data used in this study are restricted to the main diagnoses. Some of the children included in this study may have various combinations and degrees of comorbidities such as cognitive and intellectual impairment that we cannot account for in our analysis.

## 6 Conclusion

The results of this study are broadly consistent with those of other studies, and they suggest that children with a disability obtain lower GPA scores than typically developing peers without chronic conditions, and these associations are most evident for neurodevelopmental conditions. The study also shows that high-SES parents are able to compensate for their children's disability for some diagnoses but not for the diagnoses of epilepsy and autism. Given that there has been an upward trend in disabilities related to neurodevelopmental conditions, there is a need for more knowledge of how children with these disabilities can be best supported in school. Children with a low socio-economic status seems to be extra vulnerable. A particular attention should be directed to this group of children and help them to recognize and reach their full potential at school. Education creates the basis for employment opportunities and therefore important for later adult life. Intervening to prevent and treat these medical conditions and facilitate access to learning might have positive consequences on these children's educational performance.

## Ethical statement

The study was approved by the Regional Committee for Medical Research Ethics in South-East Norway. The Norwegian Data Protection Authority granted permission to access all the databases and records without written consent from the study participants.

## Declaration of competing interest

The authors declare that they have no competing interests.

## Data Availability

The authors do not have permission to share data.

## Footnotes

1 To assess eligibility for attendance benefits at different rates, NAV considers the degree of physical and psychological functional impairment, the scope of care and supervision needed, the need for stimulation, training, and physical activity, and the extent to which providing the care limits the caregiver.

## References

[bib1] Agnafors S., Barmark M., Sydsjö G. (2021). Mental health and academic performance: A study on selection and causation effects from childhood to early adulthood. Social Psychiatry and Psychiatric Epidemiology.

[bib2] Almond D., Mazumder B. (2013).

[bib3] Arnold L.E., Hodgkins P., Kahle J., Madhoo M., Kewley G. (2015). Long-term outcomes of ADHD: Academic achievement and performance. Journal of Attention Disorders.

[bib4] Ashburner J., Ziviani J., Rodger S. (2010). Surviving in the mainstream: Capacity of children with autism spectrum disorders to perform academically and regulate their emotions and behavior at school. Res. Autism Spectr. Disord..

[bib6] Beghi M., Cornaggia C.M., Frigeni B., Beghi E. (2006). Learning disorders in epilepsy. Epilepsia.

[bib7] Begum M., Chittleborough C., Pilkington R., Mittinty M., Lynch J., Penno M., Smithers L. (2020). Educational outcomes among children with type 1 diabetes: Whole-of-population linked-data study. Pediatric Diabetes.

[bib8] Bernardi F. (2012). Unequal transitions: Selection bias and the compensatory effect of social background in educational careers. Research in Social Stratification and Mobility.

[bib9] Bernardi F. (2014). Compensatory advantage as a mechanism of educational inequality:A regression discontinuity based on month of birth. Sociology of Education.

[bib10] Bernardi F., Grätz M. (2015). Making up for an unlucky month of birth in school: Causal evidence on the compensatory advantage of family background in england. Sociol. Sci..

[bib11] Bernardi F., Triventi M. (2018). Compensatory advantage in educational transitions: Trivial or substantial? A simulated scenario analysis. Acta Sociologica.

[bib12] Biedinger N. (2011). The influence of education and home environment on the cognitive outcomes of preschool children in Germany. Child Dev. Res..

[bib13] Blackburn C.M., Spencer N.J., Read J.M. (2010). Prevalence of childhood disability and the characteristics and circumstances of disabled children in the UK: Secondary analysis of the family resources survey. BMC Pediatrics.

[bib14] Blum L. (2015).

[bib15] Breen R., Jonsson J.O. (2005). Inequality of opportunity in comparative perspective: Recent research on educational attainment and social mobility. Annual Review of Sociology.

[bib16] Breslau J., Lane M., Sampson N., Kessler R.C. (2008). Mental disorders and subsequent educational attainment in a US national sample. Journal of Psychiatric Research.

[bib17] Broer M., Bai Y., Fonseca F., Broer M., Bai Y., Fonseca F. (2019). Socioeconomic inequality and educational outcomes: Evidence from twenty years of TIMSS.

[bib18] Carrilero N., Dalmau-Bueno A., García-Altés A. (2021). Socioeconomic inequalities in 29 childhood diseases: Evidence from a 1,500,000 children population retrospective study. BMC Public Health.

[bib19] Champaloux S.W., Young D.R. (2015). Childhood chronic health conditions and educational attainment: A social ecological approach. Journal of Adolescent Health.

[bib20] Chatzitheochari S., Platt L. (2019). Disability differentials in educational attainment in england: Primary and secondary effects. British Journal of Sociology.

[bib21] Cheadle J.E., Amato P.R. (2010). A quantitative assessment of lareau's qualitative conclusions about class, race, and parenting. Journal of Family Issues.

[bib22] Conley D., Lareau A. (2008).

[bib23] Cooper M.N., McNamara K.A.R., de Klerk N.H., Davis E.A., Jones T.W. (2016). School performance in children with type 1 diabetes: A contemporary population-based study. Pediatric Diabetes.

[bib24] Cooper K., Stewart K. (2021). Does household income affect children's outcomes? A systematic review of the evidence. Child Indicators Res..

[bib25] Crump C., Rivera D., London R., Landau M., Erlendson B., Rodriguez E. (2013). Chronic health conditions and school performance among children and youth. Annals of Epidemiology.

[bib26] Currie J., Stabile M. (2003). Socioeconomic status and child health: Why is the relationship stronger for older children?. The American Economic Review.

[bib27] Currie J., Stabile M. (2006). Child mental health and human capital accumulation: The case of ADHD. Journal of Health Economics.

[bib28] Currie J., Stabile M. (2007). The problems of disadvantaged youth:an economic perspective.

[bib29] Dijkhuis R., de Sonneville L., Ziermans T., Staal W., Swaab H. (2020). Autism symptoms, executive functioning and academic progress in higher education students. Journal of Autism and Developmental Disorders.

[bib30] Duncan M.J., Patte K.A., Leatherdale S.T. (2021). Mental health associations with academic performance and education behaviors in Canadian secondary school students. Canadian Journal of School Psychology.

[bib31] Eide E.R., Showalter M.H., Goldhaber D.D. (2010). The relation between children's health and academic achievement. Children and Youth Services Review.

[bib32] Esch P., Bocquet V., Pull C., Couffignal S., Lehnert T., Graas M., Fond-Harmant L., Ansseau M. (2014). The downward spiral of mental disorders and educational attainment: A systematic review on early school leaving. BMC Psychiatry.

[bib33] Evensen M., Lyngstad T.H., Melkevik O., Mykletun A. (2016). The role of internalizing and externalizing problems in adolescence for adult educational attainment: Evidence from sibling comparisons using data from the Young HUNT study. European Sociological Review.

[bib34] Fleming M., Fitton C.A., Steiner M.F.C., McLay J.S., Clark D., King A., Lindsay R.S., Mackay D.F., Pell J.P. (2019). Educational and health outcomes of children treated for type 1 diabetes: Scotland-wide record linkage study of 766,047 children. Diabetes Care.

[bib35] Fleming M., Fitton C., Steiner M., McLay J., Clark D., King A., Mackay D., Pell J. (2019). Educational and health outcomes of children treated for asthma: Scotland-wide record linkage study of 683,716 children. European Respiratory Journal.

[bib36] Fleming M., Fitton C.A., Steiner M.F.C., McLay J.S., Clark D., King A., Mackay D.F., Pell J.P. (2019). Educational and health outcomes of children and adolescents receiving antiepileptic medication: Scotland-wide record linkage study of 766 244 schoolchildren. BMC Public Health.

[bib37] Frith U. (2004). Emanuel miller lecture: Confusions and controversies about asperger syndrome. Journal of Child Psychology and Psychiatry.

[bib38] Gillies M.B., Bowen J.R., Patterson J.A., Roberts C.L., Torvaldsen S. (2018). Educational outcomes for children with cerebral palsy: A linked data cohort study. Developmental Medicine and Child Neurology.

[bib39] Guryan J., Hurst E., Kearney M. (2008). Parental education and parental time with children. The Journal of Economic Perspectives.

[bib40] Haegele J.A., Hodge S. (2016). Disability discourse: Overview and critiques of the medical and social models. Quest.

[bib41] Hibel J., Domina T., Gibbs B.G., Nunn L., Penner A. (2019). Special education and social inequality. Education and society: An introduction to key issues in the sociology of education.

[bib42] Hsin A. (2012). Is biology destiny? Birth weight and differential parental treatment. Demography.

[bib43] Huang M.-H. (2020). Compensatory advantage and the use of out-of-school-time tutorials: A cross-national study. Research in Social Stratification and Mobility.

[bib45] Jangmo A., Stålhandske A., Chang Z., Chen Q., Almqvist C., Feldman I., Bulik C.M., Lichtenstein P., D'Onofrio B., Kuja-Halkola R. (2019). Attention-deficit/hyperactivity disorder, school performance, and effect of medication. Journal of the American Academy of Child & Adolescent Psychiatry.

[bib46] Jarl J., Alriksson-Schmidt A. (2021). School outcomes of adolescents with cerebral palsy in Sweden. Developmental Medicine and Child Neurology.

[bib47] Jensen M.R., van der Wel K.A., Bråthen M. (2021). Adolescent mental health disorders and upper secondary school completion – the role of family resources. Scandinavian Journal of Educational Research.

[bib48] Keen D., Webster A., Ridley G. (2015). How well are children with autism spectrum disorder doing academically at school? An overview of the literature. Autism : The International Journal of Research and Practice.

[bib49] Keilow M., Holm A., Fallesen P. (2018). Medical treatment of Attention Deficit/Hyperactivity Disorder (ADHD) and children's academic performance. PLoS One.

[bib50] Kim S.H., Bal V.H., Lord C. (2018). Longitudinal follow-up of academic achievement in children with autism from age 2 to 18. Journal of Child Psychology and Psychiatry.

[bib51] Klein M., Sosu E.M., Dare S. (2022). School absenteeism and academic achievement: Does the reason for absence matter?. AERA Open.

[bib52] Koegel L.K., Vernon T., Koegel R.L., Koegel B.L., Paullin A.W. (2012). Improving social engagement and initiations between children with autism spectrum disorder and their peers in inclusive settings. Journal of Positive Behavior Interventions.

[bib53] Kurowski M., Černý M., Trapl F. (2022). A review study of research articles on the barriers to inclusive education in primary schools. Journal on Efficiency and Responsib. Edu. Sci..

[bib54] Kvande M.N., Belsky J., Wichstrøm L. (2018). Selection for special education services: The role of gender and socio-economic status. European Journal of Special Needs Education.

[bib55] Lareau A. (1989).

[bib56] Lareau A. (2011).

[bib57] Lereya S.T., Patel M., Dos Santos J., Deighton J. (2019). Mental health difficulties, attainment and attendance: A cross-sectional study. European Child & Adolescent Psychiatry.

[bib58] Lundholm C., Brew B.K., D'Onofrio B.M., Osvald E.C., Larsson H., Almqvist C. (2020). Asthma and subsequent school performance at age 15-16 years: A Swedish population-based sibling control study. Scientific Reports.

[bib59] van der Mark E., Conradie I., Dedding C., Broerse J. (2017). How poverty shapes caring for a disabled child: A narrative literature review. Journal of International Development.

[bib60] Mathiesen I.H., Vedøy G. (2012). Stavanger.

[bib61] McKinley Yoder C.L., Cantrell M.A. (2019). Childhood disability and educational outcomes: A systematic review. Journal of Pediatric Nursing.

[bib62] Melkevik O., Nilsen W., Evensen M., Reneflot A., Mykletun A. (2016). Internalizing disorders as risk factors for early school leaving: A systematic review. Adolesc. Res. Rev..

[bib63] Mikkonen J., Remes H., Moustgaard H., Martikainen P. (2020). Evaluating the role of parental education and adolescent health problems in educational attainment. Demography.

[bib64] Mitchell R.J., McMaugh A., Homaira N., Lystad R.P., Badgery-Parker T., Cameron C.M. (2022). The impact of childhood asthma on academic performance: A matched population-based cohort study. Clinical and Experimental Allergy.

[bib65] Mizunoya S., Mitra S., Yamasaki I. (2018). Disability and school attendance in 15 low- and middle-income countries. World Development.

[bib66] Nilsson S., Ödling M., Andersson N., Bergström A., Kull I. (2018). Does asthma affect school performance in adolescents? Results from the Swedish population-based birth cohort BAMSE. Pediatric Allergy & Immunology.

[bib68] Nordmo M., Kinge J.M., Reme B.-A., Flatø M., Surén P., Wörn J., Magnus P., Stoltenberg C., Torvik F.A. (2022). The educational burden of disease: A cohort study. The Lancet Public Health.

[bib67] Noble KG, Houston SM, Brito NH, Bartsch H, Kan E, Kuperman JM, Akshoomoff N, Amaral DG, Bloss CS, Libiger O, Schork NJ, Murray SS, Casey BJ, Chang L, Ernst TM, Frazier JA, Gruen JR, Kennedy DN, Van Zijl P, Mostofsky S, Kaufmann WE, Kenet T, Dale AM, Jernigan TL, Sowell ER (2015). Family income, parental education and brain structure in children and adolescents. Nature Neuroscience.

[bib69] O'Brien G., Pearson J. (2004). Autism and learning disability. Autism.

[bib70] Parcel T.L., Dufur M.J., Zito R.C. (2010). Capital at home and at school: A review and synthesis. Journal of Marriage and Family.

[bib71] Persson E., Persson S., Gerdtham U.-G., Steen Carlsson K. (2019). Effect of type 1 diabetes on school performance in a dynamic world: New analysis exploring Swedish register data. Applied Economics.

[bib72] Rasalingam A., Brekke I., Dahl E., Helseth S. (2021). Impact of growing up with somatic long-term health challenges on school completion, NEET status and disability pension: A population-based longitudinal study. BMC Public Health.

[bib73] Reiss F. (2013). Socioeconomic inequalities and mental health problems in children and adolescents: A systematic review. Social Science & Medicine.

[bib74] Rivera LA, Tilcsik A: Not in my schoolyard: Disability discrimination in educational access. American Sociological Review, 0(0):00031224221150433.

[bib75] Schneider T. (2020). Asthma and academic performance among children and youth in north America: A systematic review. Journal of School Health.

[bib76] Senter J.P., Smith B.M., Prichett L.M., Connor K.A., Johnson S.B. (2021). Pediatric asthma is associated with poorer 3-year academic achievement in urban elementary and middle-school students. Academic Pediatrics.

[bib77] Shandra C.L., Hogan D.P. (2009). The educational attainment process among adolescents with disabilities and children of parents with disabilities. International Journal of Disability, Development and Education.

[bib78] von Simson K., Brekke I., Hardoy I. (2020). The impact on educational attainment of mental health problems in adolescence. The Journal of Educational Research.

[bib79] Sosu E.M., Dare S., Goodfellow C., Klein M. (2021). Socioeconomic status and school absenteeism: A systematic review and narrative synthesis. The Review of Education.

[bib80] Sunde H.F., Kleppestø T.H., Gustavson K., Nordmo M., Reme B.-A., Torvik F.A. (2022). The ADHD deficit in school performance across sex and parental education: A prospective sibling-comparison register study of 344,152 Norwegian adolescents. JCPP Advances.

[bib81] The Norwegian Directorate for Education and Training (2019).

[bib83] Veldman K., Bültmann U., Stewart R.E., Ormel J., Verhulst F.C., Reijneveld S.A. (2014). Mental health problems and educational attainment in adolescence: 9-Year follow-up of the TRAILS study. PLoS One.

