# Supplementary material for: Educational achievement among children with a disability: do parental resources compensate for disadvantage?
Source: SSM Popul Health. 2023 Jul 17;23:101465. doi: 10.1016/j.ssmph.2023.101465 (PMC10404540; doi:10.1016/j.ssmph.2023.101465)
Supplement: Multimedia component 1 [file mmc1.docx]

Appendix: Figure 1, density plot of GPA score by diagnosis for our sample


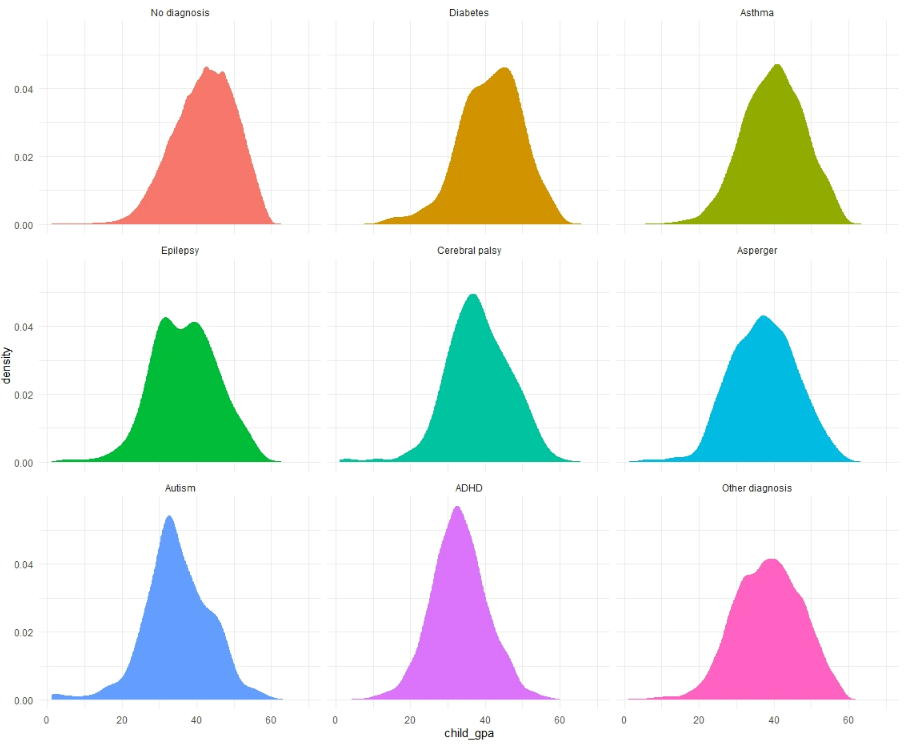


Appendix: Table 1. Dependent variable No registered GPA Score =1, registered GPA score=0. Model 3 includes school FE. Linear probability model

|  | (1) | (2) | (3) |
| --- | --- | --- | --- |
|  | Bivariate | Adjusted | Adjusted+FE |
| No diagnosis (ref) |  |  |  |
|  |  |  |  |
| Diabetes | -0.292^***^ | -0.279^***^ | -0.259^***^ |
|  | (0.012) | (0.017) | (0.017) |
| Asthma | -0.152^***^ | -0.142^***^ | -0.133^***^ |
|  | (0.007) | (0.011) | (0.010) |
| Epilepsy | 0.015 | 0.020 | 0.018 |
|  | (0.012) | (0.025) | (0.024) |
| Cerebral palsy | 0.094^***^ | 0.097^***^ | 0.077^**^ |
|  | (0.012) | (0.026) | (0.026) |
| Asperger | -0.058^***^ | -0.048^*^ | -0.039^*^ |
|  | (0.009) | (0.020) | (0.019) |
| Autism | 0.240^***^ | 0.243^***^ | 0.227^***^ |
|  | (0.010) | (0.021) | (0.021) |
| ADHD | -0.136^***^ | -0.132^***^ | -0.119^***^ |
|  | (0.006) | (0.011) | (0.011) |
| Other diagnosis | -0.005 | 0.001 | -0.006 |
|  | (0.005) | (0.009) | (0.009) |
| No attendance benefit (ref) |  |  |  |
|  |  |  |  |
| Low attendance benefit | 0.122^***^ | 0.119^***^ | 0.114^***^ |
|  | (0.006) | (0.010) | (0.010) |
| High attendance benefit | 0.348^***^ | 0.344^***^ | 0.314^***^ |
|  | (0.006) | (0.010) | (0.010) |
| Boys (ref) |  |  |  |
|  |  |  |  |
| Girls |  | -0.001 | -0.001^*^ |
|  |  | (0.001) | (0.001) |
| Upper secondary school and lower (ref) |  | 0.000 | 0.000 |
| Bachelor’s degree level and above |  | -0.004^***^ | -0.011^***^ |
|  |  | (0.001) | (0.001) |
| Unknown education |  | 0.045^***^ | 0.056^***^ |
|  |  | (0.002) | (0.002) |
| ≥ median income (ref) |  |  |  |
|  |  |  |  |
| <median income |  | 0.003^***^ | 0.005^***^ |
|  |  | (0.001) | (0.001) |
| Not married (ref) |  |  |  |
|  |  |  |  |
| Married/Registered partner |  | -0.017^***^ | -0.021^***^ |
|  |  | (0.001) | (0.001) |
| Divorced/separated |  | -0.006^***^ | -0.011^***^ |
|  |  | (0.001) | (0.001) |
| Majority (ref) |  |  |  |
| Immigrant background |  | 0.025^***^ | 0.027^***^ |
|  |  | (0.001) | (0.001) |
| Mother age at birth |  | -0.008^***^ | -0.005^***^ |
|  |  | (0.001) | (0.001) |
| Mother age at birth squared |  | 0.000^***^ | 0.000^***^ |
| No siblings (ref) |  |  |  |
|  |  |  |  |
| 1 sibling |  | -0.007^***^ | -0.006^***^ |
|  |  | (0.002) | (0.002) |
| 2 or more siblings |  | -0.002 | -0.000 |
|  |  | (0.002) | (0.002) |
| Constant | 0.032^***^ | 0.153^***^ | 0.116^***^ |
|  | (0.000) | (0.011) | (0.011) |
| Observations | 326874 | 326874 | 307249 |
| *R*^2^ | 0.100 | 0.117 | 0.248 |
| Adjusted *R*^2^ | 0.100 | 0.117 | 0.244 |

Last model has school FE. All models are controlled for birth cohort and robust SE.

^*^ *p* < 0.05, ^**^ *p* < 0.01, ^***^ *p* < 0.001

Appendix Table 2: Descriptive statistics of variables used in analysis by child diagnoses

| Variables | 'Typically developing children, (n = 278.621) | Diabetes, (n = 309) | Asthma, (n = 1496) | Epilepsy, N = 229 | Cerebral palsy, (n = 191) | Asperger, (n = 486) | Autism, (n = 221) | ADHD, (n = 3126) | Other diagnosis, (n = 8023) |
| --- | --- | --- | --- | --- | --- | --- | --- | --- | --- |
| GPA score, mean (SD) | 42.20(8.19) | 41.60 (8.18) | 40.05 (8.14) | 36.93 (8.48) | 38.25 (8.31) | 37.30 (8.61) | 34.82 (8.78) | 33.13 (7.49) | 38.53 (8.91) |
| Exemption from GPA (%) | 3.29 | 6.61 | 6.30 | 35.0 | 43.0 | 30.0 | 61.0 | 21.21 | 28.6 |
| Girls (%) | 49.72 | 50.49 | 36.43 | 51.97 | 38.74 | 24.69 | 20.36 | 29.56 | 40.72 |
| Immigrant background, child (%) | 14.29 | 5.83 | 6.62 | 10.92 | 12.04 | 4.94 | 14.93 | 3.23 | 8.90 |
| Parents upper secondary school and lower (%) | 35.97 | 40.45 | 44.39 | 36.24 | 39.27 | 39.71 | 36.65 | 48.82 | 41.92 |
| Parents bachelor’s degree level and above (%) | 54.15 | 54.37 | 47.66 | 54,59 | 54.45 | 53.70 | 54.75 | 37.68 | 48.97 |
| Parents unknown education (%) | 9.88 | 5.18 | 7.95 | 9.17 | 6.28 | 6.58 | 6.28 | 13.50 | 9.11 |
| Parental income < median (%) | 44.72 | 46.28 | 52.81 | 48.03 | 48.69 | 60.70 | 60.63 | 64.46 | 54.44 |
| Parental income ≥ median (%) | 55.78 | 53.72 | 47.19 | 51.97 | 51.13 | 39.30 | 39.37 | 35.54 | 45.56 |
| Mother age at birth, mean (SD | 29.74 (5.15) | 30.0 (4.67) | 29.51 (4.76) | 29.34 (5.48) | 29.32 (4.82) | 29.06 (5.34) | 29.85 (5.53) | 28.15 (5.32) | 29.45 (5.14) |
| Marital status  Married (%) | 27.30 | 22.98 | 22.46 | 22.27 | 24.61 | 27.37 | 24.89 | 33.65 | 26.42 |
| Married/registered partner (%) | 60.55 | 66.34 | 59.56 | 61.57 | 62.30 | 59.05 | 60.18 | 47.27 | 56.94 |
| Divorced (%) | 12.15 | 10.68 | 17.98 | 16.16 | 13.09 | 13.58 | 14.93 | 19.10 | 16.64 |
| no siblings (%) | 7.21 | 7.44 | 5.08 | 5.68 | 7.85 | 10.49 | 8.14 | 8.09 | 6.84 |
| 1 siblings (%) | 42.04 | 44.98 | 38.03 | 39.30 | 48.17 | 42.39 | 37.10 | 40.44 | 39.14 |
| 2 or more (%) siblings | 50.75 | 47.57 | 56.89 | 55.02 | 43.98 | 47.12 | 54.75 | 51.47 | 54.02 |

Appendix: Table 3. Dependent variable GPA Score. Interaction term between child disability and parents's highest level of education (model4). OLS, school-level fixed effects in model 3 and 4.

|  | (1) | (2) | (3) | (4) |
| --- | --- | --- | --- | --- |
|  | Bivariate | Adjusted | Adjusted+FE | Adjusted+FE+interaction |
| 'Typically developing children, no diagnosis (ref) |  |  |  |  |
|  |  |  |  |  |
| Diabetes | -0.664 | 2.325^***^ | 2.210^***^ |  |
|  | (0.454) | (0.464) | (0.457) |  |
| Asthma | -2.016^***^ | 1.269^***^ | 1.218^***^ |  |
|  | (0.208) | (0.287) | (0.282) |  |
| Epilepsy | -4.992^***^ | -2.164^***^ | -2.101^***^ |  |
|  | (0.548) | (0.598) | (0.585) |  |
| Cerebral palsy | -3.751^***^ | -0.414 | -0.403 |  |
|  | (0.591) | (0.584) | (0.576) |  |
| Asperger | -4.654^***^ | -0.194 | -0.159 |  |
|  | (0.382) | (0.442) | (0.435) |  |
| Autism | -7.166^***^ | -2.506^***^ | -2.591^***^ |  |
|  | (0.586) | (0.647) | (0.639) |  |
| ADHD | -8.700^***^ | -3.504^***^ | -3.490^***^ |  |
|  | (0.132) | (0.271) | (0.266) |  |
| Other diagnosis | -3.422^***^ | -0.092 | -0.100 |  |
|  | (0.099) | (0.228) | (0.225) |  |
| No attendance benefit (ref) |  |  |  |  |
|  |  |  |  |  |
| Low attendance benefit |  | -2.319^***^ | -2.182^***^ | -2.193^***^ |
|  |  | (0.248) | (0.244) | (0.243) |
| High attendance benefit |  | -3.452^***^ | -3.326^***^ | -3.323^***^ |
|  |  | (0.243) | (0.240) | (0.239) |
| Boys (ref) |  |  |  |  |
| Girls |  | 4.369^***^ | 4.368^***^ | 4.367^***^ |
|  |  | (0.026) | (0.026) | (0.026) |
| Upper secondary school and lower (ref) |  |  |  |  |
| Bachelor’s degree level and above |  | 4.203^***^ | 4.070^***^ | 4.106^***^ |
|  |  | (0.030) | (0.031) | (0.031) |
| Unknown education |  | -1.992^***^ | -1.992^***^ | -1.957^***^ |
|  |  | (0.057) | (0.056) | (0.058) |
| <median income (ref) |  |  |  |  |
|  |  |  |  |  |
| ≥median income |  | 1.458^***^ | 1.403^***^ | 1.401^***^ |
|  |  | (0.029) | (0.029) | (0.029) |
| Not married (ref) |  |  |  |  |
|  |  |  |  |  |
| Married/Registered partner |  | 1.461^***^ | 1.436^***^ | 1.437^***^ |
|  |  | (0.033) | (0.033) | (0.033) |
| Divorced/separated |  | -0.454^***^ | -0.417^***^ | -0.416^***^ |
|  |  | (0.047) | (0.047) | (0.047) |
| Majority (ref) |  |  |  |  |
|  |  |  |  |  |
| Immigrant background |  | -1.029^***^ | -1.050^***^ | -1.048^***^ |
|  |  | (0.046) | (0.048) | (0.048) |
| Mother age at birth |  | 0.679^***^ | 0.673^***^ | 0.674^***^ |
|  |  | (0.024) | (0.024) | (0.024) |
| Mother age at birth squared |  | -0.009^***^ | -0.009^***^ | -0.009^***^ |
| No siblings (ref) |  |  |  |  |
|  |  |  |  |  |
| 1 sibling |  | -0.026 | -0.000 | -0.001 |
|  |  | (0.056) | (0.055) | (0.055) |
| 2 or more siblings |  | -0.740^***^ | -0.694^***^ | -0.695^***^ |
|  |  | (0.055) | (0.055) | (0.055) |
| **Interaction term, Child disability *parental education** |  |  |  |  |
| Diabetes*Upper secondary school and lower |  |  |  | 2.695^***^ |
|  |  |  |  | (0.691) |
| Diabetes*Bachelor’s degree level and above |  |  |  | 2.045^***^ |
|  |  |  |  | (0.537) |
| Diabetes*Unknown education |  |  |  | 0.130 |
|  |  |  |  | (2.350) |
| Asthma*Upper secondary school and lower |  |  |  | 1.533^***^ |
|  |  |  |  | (0.357) |
| Asthma*Bachelor’s degree level and above |  |  |  | 1.056^**^ |
|  |  |  |  | (0.334) |
| Asthma*Unknown education |  |  |  | 0.535 |
|  |  |  |  | (0.820) |
| Epilepsy*Upper secondary school and lower |  |  |  | -0.042 |
|  |  |  |  | (0.881) |
| Epilepsy*Bachelor’s degree level and above |  |  |  | -3.691^***^ |
|  |  |  |  | (0.751) |
| Epilepsy*Unknown education |  |  |  | -0.778 |
|  |  |  |  | (1.870) |
| Cerebral palsy*Upper secondary school and lower |  |  |  | -0.173 |
|  |  |  |  | (0.928) |
| Cerebral palsy*Bachelor’s degree level and above |  |  |  | -0.760 |
|  |  |  |  | (0.730) |
| Cerebral palsy*Unknown education |  |  |  | 1.283 |
|  |  |  |  | (1.635) |
| Asperger*Upper secondary school and lower |  |  |  | -0.046 |
|  |  |  |  | (0.585) |
| Asperger*Bachelor’s degree level and above |  |  |  | -0.625 |
|  |  |  |  | (0.572) |
| Asperger*Unknown education |  |  |  | 2.927^*^ |
|  |  |  |  | (1.442) |
| Autism*Upper secondary school and lower |  |  |  | -0.529 |
|  |  |  |  | (1.009) |
| Autism*Bachelor’s degree level and above |  |  |  | -4.111^***^ |
|  |  |  |  | (0.791) |
| Autism*Unknown education |  |  |  | -1.749 |
|  |  |  |  | (2.331) |
| ADHD*Upper secondary school and lower |  |  |  | -3.033^***^ |
|  |  |  |  | (0.292) |
| ADHD*Bachelor’s degree level and above |  |  |  | -4.398^***^ |
|  |  |  |  | (0.317) |
| ADHD*Unknown education |  |  |  | -2.582^***^ |
|  |  |  |  | (0.422) |
| Other diagnosis* Upper secondary school and lower |  |  |  | 0.210 |
|  |  |  |  | (0.247) |
| Other diagnosis*Bachelor’s degree level and above |  |  |  | -0.160 |
|  |  |  |  | (0.241) |
| Other diagnosis*Unknown education |  |  |  | -1.159^**^ |
|  |  |  |  | (0.380) |
| Constant | 42.196^***^ | 24.680^***^ | 25.054^***^ | 25.017^***^ |
|  | (0.015) | (0.360) | (0.356) | (0.356) |
| Observations | 292702 | 292702 | 292702 | 292702 |
| *R*^2^ | 0.071 | 0.259 | 0.285 | 0.285 |
| Adjusted *R*^2^ | 0.067 | 0.259 | 0.281 | 0.282 |

Robust SE. All models are controlled for birth cohort.

^*^ *p* < 0.05, ^**^ *p* < 0.01, ^***^ *p* < 0.001

Appendix: Table 4. Dependent variable GPA Score. Interaction term between child disability and parents's income. OLS, school-level fixed effects.

|  | (1) |
| --- | --- |
|  | Adjusted+FE+interaction |
| *Typically developing children* , no diagnosis (ref) |  |
| Diabetes * < median income | 1.545^*^ |
|  | (0.647) |
| Diabetes * ≥ median income | 2.779^***^ |
|  | (0.560) |
| Asthma * < median income | 1.027^**^ |
|  | (0.339) |
| Asthma * ≥ median income | 1.433^***^ |
|  | (0.340) |
| Epilepsy * < median income | -1.708^*^ |
|  | (0.752) |
| Epilepsy * ≥ median income | -2.467^**^ |
|  | (0.828) |
| Cerebral palsy * < median income | -0.040 |
|  | (0.814) |
| Cerebral palsy * ≥ median income | -0.748 |
|  | (0.749) |
| Asperger * < median income | 0.281 |
|  | (0.532) |
| Asperger * ≥ median income | -0.841 |
|  | (0.614) |
| Autism * < median income | -1.950^*^ |
|  | (0.824) |
| Autism * ≥ median income | -3.581^***^ |
|  | (0.913) |
| ADHD * < median income | -3.078^***^ |
|  | (0.284) |
| ADHD * ≥ median income | -4.236^***^ |
|  | (0.312) |
| Other diagnosis * < median income | -0.109 |
|  | (0.241) |
| Other diagnosis * ≥ median income | -0.089 |
|  | (0.243) |
| No attendance benefit | 0.000 |
|  | (.) |
| Low attendance benefit | -2.182^***^ |
|  | (0.244) |
| High attendance benefit | -3.324^***^ |
|  | (0.240) |
| Boys (ref) |  |
|  |  |
| Girls | 4.368^***^ |
|  | (0.026) |
| Upper secondary school and lower (ref) | 0.000 |
|  | (.) |
| Bachelor’s degree level and above | 4.069^***^ |
|  | (0.031) |
| Unknown education | -1.994^***^ |
|  | (0.056) |
| < median income (ref) |  |
|  |  |
| ≥ median income | 1.415^***^ |
|  | (0.030) |
| Not married (ref) |  |
|  |  |
| Married/Registered partner | 1.436^***^ |
|  | (0.033) |
| Divorced/separated | -0.417^***^ |
|  | (0.047) |
| Majority (ref) |  |
|  |  |
| Immigrant background | -1.047^***^ |
|  | (0.048) |
| Mother age at birth | 0.674^***^ |
|  | (0.024) |
| Mother age at birth squared | -0.009^***^ |
|  | (0.000) |
| No siblings (ref) |  |
|  |  |
| 1 sibling | -0.002 |
|  | (0.055) |
| 2 or more siblings | -0.695^***^ |
|  | (0.055) |
| Constant | 25.032^***^ |
|  | (0.356) |
| Observations | 292702 |
| *R*^2^ | 0.285 |
| Adjusted *R*^2^ | 0.282 |

Robust SE, controlled for birth cohort.

^*^ *p* < 0.05, ^**^ *p* < 0.01, ^***^ *p* < 0.001
